# Understanding the Columnar Lined Esophagus and Its Variations in Length With Age: A Cadaveric Study

**DOI:** 10.7759/cureus.46095

**Published:** 2023-09-27

**Authors:** Sudipta D Baruah, Satyajit Mitra, Bishwajeet Saikia, Joydev Sarma, Monalisa Nath

**Affiliations:** 1 Data Labelling, KaliberAI, Guwahati, IND; 2 Anatomy, Gauhati Medical College, Guwahati, IND; 3 Anatomy, North Eastern Indira Gandhi Regional Institute of Health and Medical Sciences (NEIGRIHMS), Shillong, IND

**Keywords:** lower end of esophagus, anatomy, histology, columnar lined esophagus, esophagus

## Abstract

Introduction

Columnar lined epithelium (CLE) of the esophagus holds particular importance in diagnosing Barrett's esophagus (BE). In Asia, the prevalence of BE ranges from 0.06% to 6.2%. In India, prevalence estimates vary from 2.6% to 23%. The frequency of esophageal adenocarcinoma has also been observed to be increasing alarmingly over the past few decades. The length of CLE as a criterion for diagnosing BE is the subject of considerable debate. Changes in CLE length among different age groups may exist. Our study aims to measure the length of CLE, or the distance between the angle of His and the Z Line (AZ distance), in normal individuals from Northeast India, and to analyze its variation across different age groups.

Materials and methods

The study was conducted in the Department of Anatomy and the Department of Forensic Medicine and Toxicology at Gauhati Medical College, Guwahati, Assam, India, during the period 2017-2019. Once opened, each specimen was laid flat on a board. The distance between the A and Z lines was measured using a pair of vernier calipers. This distance was recorded as the AZ distance in millimeters (mm).

Results

The mean AZ Distance was found to be 12.4 ± 5.3 mm. A significant correlation between age and AZ distance was observed.

Conclusion

Our present study suggests that the length of the CLE increases with age. This observation offers an opportunity to revisit or revise the diagnostic criteria based on CLE length, taking into account the age of the individual.

## Introduction

In the mid-1930s, the lower end of the esophagus and the gastric cardia grabbed the attention of surgeons. The reason for this increased attention was the presence of numerous anatomical transitions, the most significant of which is the stratified to columnar transition of the epithelium. This columnar lined epithelium (CLE) of the esophagus is of particular importance while diagnosing Barrett’s esophagus (BE). People with gastroesophageal reflux disease (GERD) are at high risk of developing BE [1]. In Asia, excluding Japan, the prevalence of BE ranges from 0.06% to 6.2% [1]; in Japan, the prevalence ranges from 19.9% [2] to 43% [3]. The prevalence in India is estimated to range from 2.6% to 23% [4,5]. The frequency of esophageal adenocarcinoma, of which BE is often the precursor, has also been seen to increase alarmingly over the past few decades [6,7].

## Materials and methods

Relevant anatomy of the gastroesophageal junction

As the esophagus joins the stomach, its lumen becomes continuous. This part of the esophagus, the abdominal esophagus (0.5-2.5 cm) [8], is intraperitoneal and meets the cardiac end of the stomach, creating the gastroesophageal junction (GEJ).

Definition of CLE

Anatomical Examination

**Figure 1:**
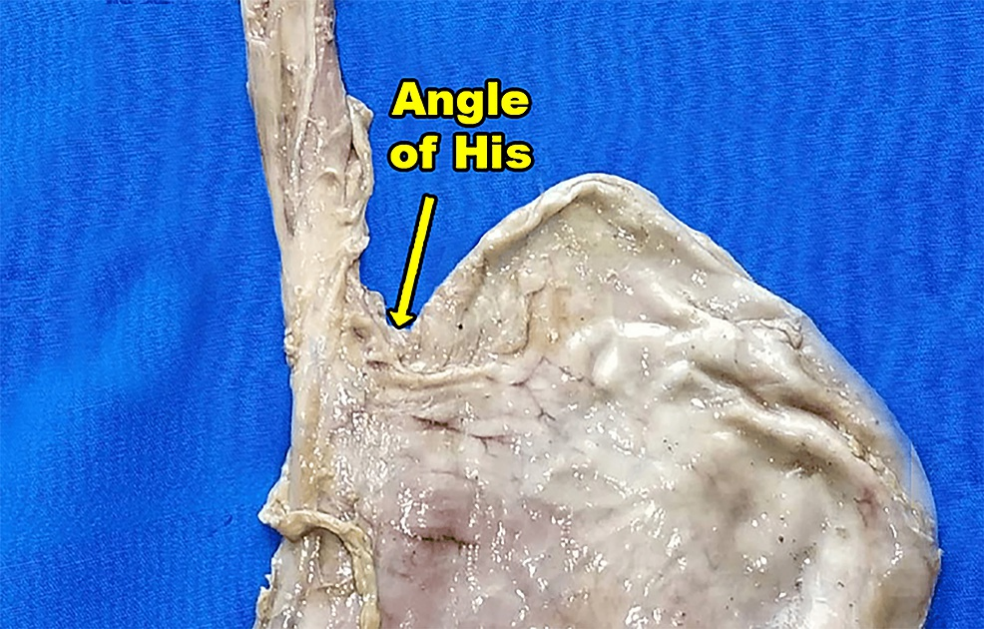
A specimen of esophagus and stomach selected for the research.

**Figure 2:**
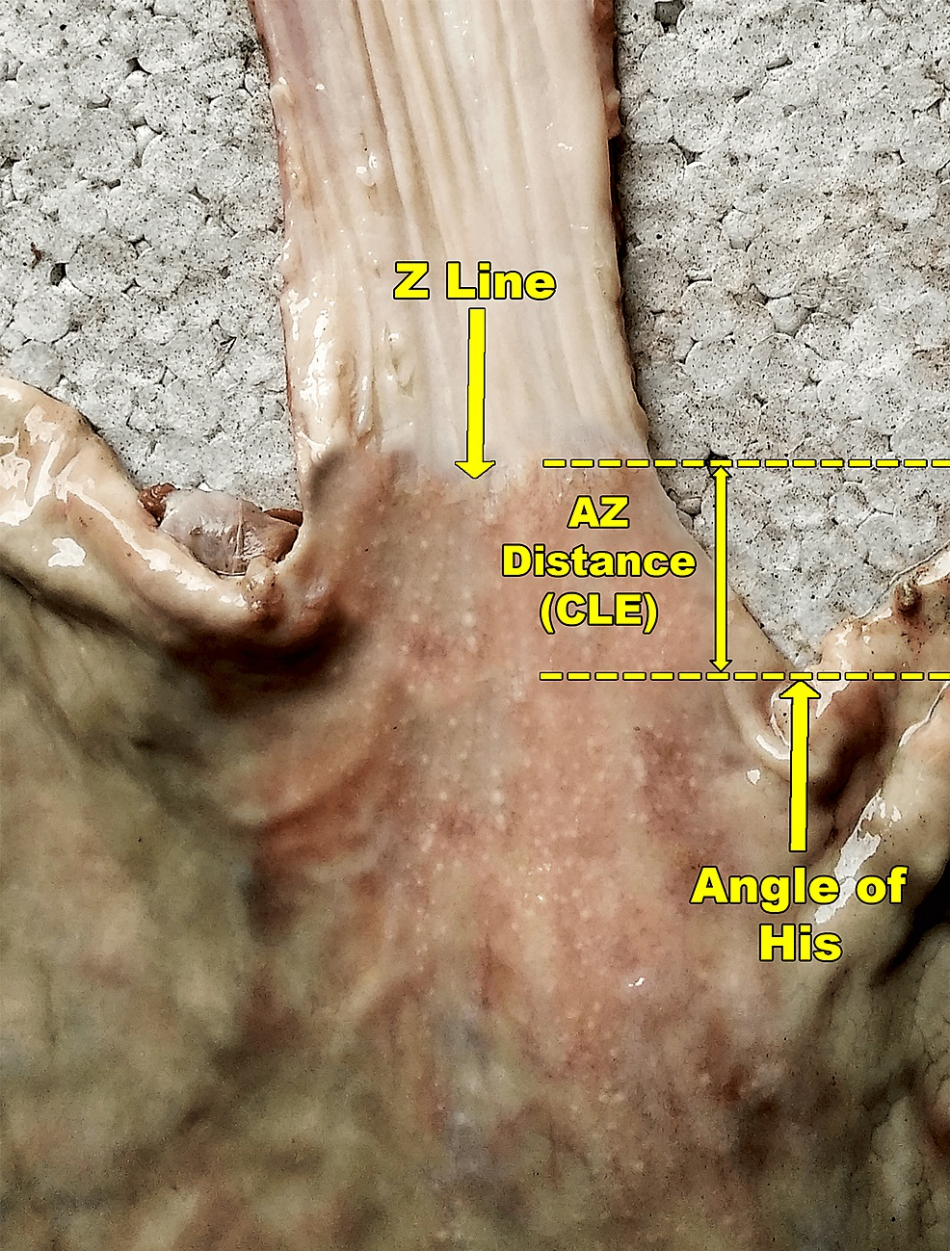
A cut specimen of esophagus and stomach showing the gross anatomical landmarks (stretched on the right side).

Externally, this is marked by the cardiac notch of the stomach or the angle of His (A). The A corresponds to the anatomical GEJ [9]. On gross anatomical examination, the squamocolumnar junction is visible internally as a distinct jagged margin, which is popularly called the Z line [2]. Until this point, the proximal esophagus is lined by a stratified squamous non-keratinised epithelium. The Z line is usually a little distance proximal to the A [10]. This distance of the Z line from the A, which can be termed the AZ distance, represents the terminal part of the esophagus, lined by simple columnar epithelium, and thus corresponds to the CLE (Figures 1, 2).

Endoscopic Examination

**Figure 3:**
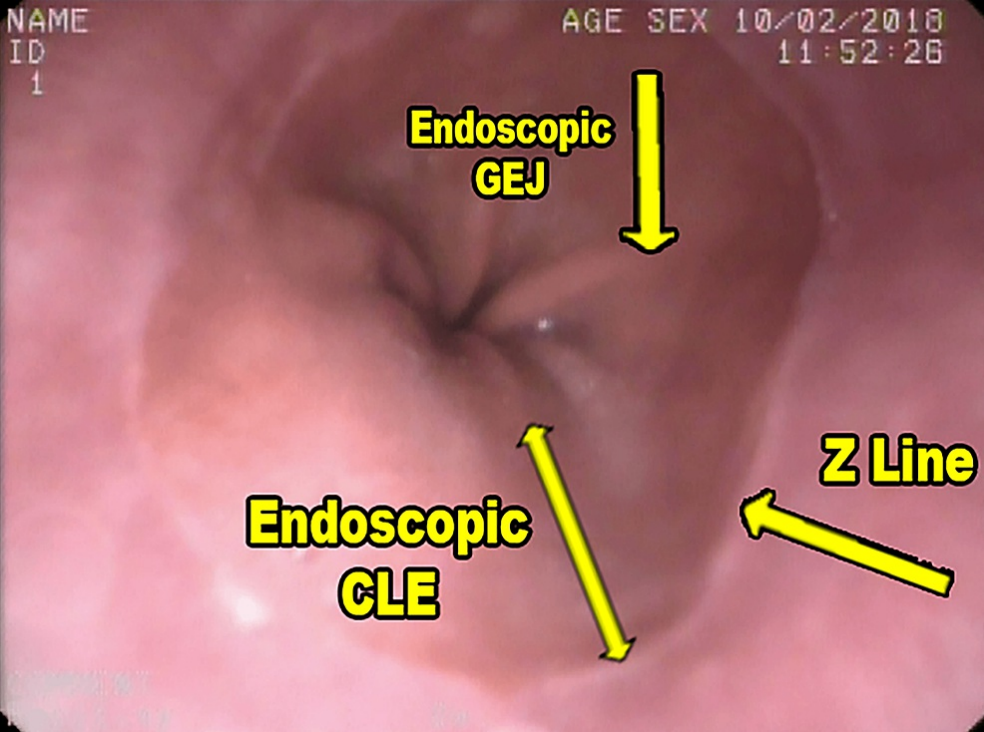
Endoscopic landmarks for determining the CLE. Endoscopic picture provided by Dr. PKD Phukan, General Surgeon. CLE: columnar lined epithelium.

During upper gastrointestinal endoscopy, the Z line is visible as a junction between the pale pearly colored squamous epithelium and the salmon-colored columnar epithelium. The GEJ is usually identified as a pinch at the distal end of the esophagus, coinciding with the most proximal appearance of the gastric rugal folds. This point marks the beginning of the stomach and its distance from the Z line conventionally corresponds to the CLE [4,11]. Endoscopically the AZ distance/length of CLE can be measured by subtracting the distance of the incisor to the squamocolumnar junction (Z line) from the distance of the incisor to the gastroesophageal junction (Figure 3).

The distribution of the AZ distance/length of CLE is a very significant and well-established criterion for diagnosing BE in clinical practice. The length of CLE as a criterion for diagnosing BE surrounds much controversy and has been kept arbitrary. The CLE can be expected to have a length of up to 2 cm in normal individuals [12,13]. Later on, more views came in where the acceptable value of CLE length to be diagnosed as BE was kept arbitrarily at >3 cm [14]. There were many more studies challenging the previous ones, creating controversies that still surround the normal CLE length. It may also be worth mentioning here that there may be changes in the CLE length among the different age groups that can be very critical in diagnosing an abnormal proximal shift in the Z line, indicative of BE. Therefore, with all of these arbitral values that surround the CLE length, our study attempted to measure the length of the CLE/AZ distance in normal individuals from North East India and to analyze its variation across different age groups.

The study was conducted in a tertiary care institution during the period of 2017-2019. Ethical clearance was obtained from the Institutional Ethics Committee of Gauhati Medical College and Hospital (approval MC/190/2007/Pt-1/EC/134). The specimens were collected during the routine dissection of cadavers in the Department of Anatomy, and consecutive specimens were obtained from the autopsy. A total of 50 specimens were collected, of which 32 were from male and 18 were from female cadavers. The inclusion and exclusion criteria are given in Table 1. 

**Table 1:** Inclusion and exclusion criteria.

Inclusion Criteria	Exclusion Criteria
Road traffic accident without thoracoabdominal trauma	Death due to poisoning
Death due to cardiorespiratory failure	Evidence of previous gastrointestinal surgery
Death due to cerebral stroke	Road traffic accident involving thorax and abdomen
Death due to any other cause that does not affect the esophagus and stomach	History of any condition that could possibly affect the esophagus and stomach like gastroesophageal reflux disease, frequent gastritis, acid peptic disease, etc.

The autopsies were performed within 48 hours after death. The stomach and the esophagus were preserved by placing them in a container with 10% formalin. The lower two-thirds of the esophagus and the stomach were removed for the purpose of the study. The stomach was cut open from the pylorus along the greater curvature. The incision was extended to the esophagus [15]. Once opened, the specimen was laid flat on a board. The angle of His (A) was noted as the point of deepest indentation between the esophagus and the stomach. The Z line was noted as the zig-zag line demarcating the junction between the non-keratinized stratified squamous epithelium above and the simple columnar epithelium below. The distance between the A and the Z line was measured by using a pair of vernier calipers. This distance was noted as AZ in millimeters (mm) [16].

Statistical tools

The data were analyzed to find out the mean, standard deviation, and standard error of the mean. The age of the individual was obtained from the death certificate. The correlation of the length of the columnar-lined esophagus with age was analyzed using Pearson’s product-moment correlation. IBM SPSS Statistics for Windows, Version 25 (Released 2017; IBM Corp., Armonk, New York, United States) was used for the analysis of data.

## Results

Of the 50 specimens that were studied, 18 specimens were females and 32 were males. The data were collected in age groups spanning 20 years each (Table 2).

**Table 2:** Age-wise and gender-wise distribution of the specimen to study the morphology and histology of the gastroesophageal junctions.

Age Groups	Male	Female
0-20 years	5	2
20-40 years	15	9
40-60 years	9	6
60 years and above	3	1
Total	32	18
Grand total	50	

The mean and standard deviation of the AZ distance for each age group are shown in Table 3.

**Table 3:** Mean and standard deviation (in mm) of the AZ distance in various age groups. AZ distance: distance from the angle of His to the Z line.

Age group	Mean (mm)
0-20	5.3666 ± 5.5174
20-40	12.636 ± 5.025
40-60	13.72 ± 3.724
Above 60	17.025 ± 1.9972

The mean AZ distance was found to be 12.4 mm with a standard deviation of 5.3 mm. The standard error of the mean was found to be 0.75 mm. For testing the correlation between age and AZ distance, Pearson’s product-moment correlation measure was used.

The null hypothesis is as follows: H0: There is no correlation between age and AZ distance.

**Figure 4:**
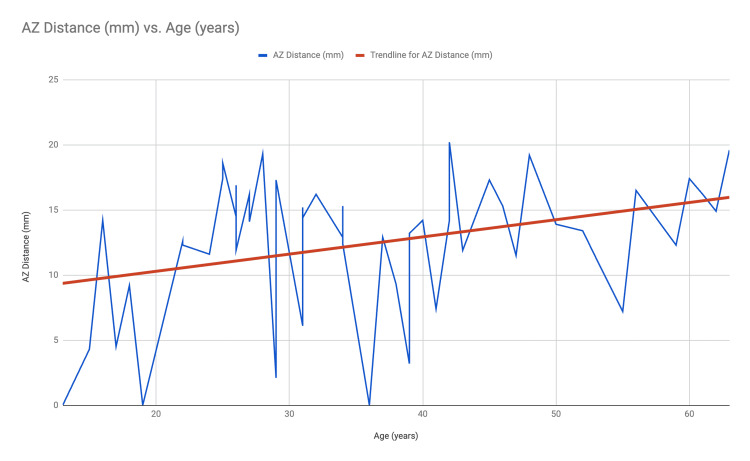
Line graph with a trendline showing the AZ distance (in mm) against age.

The value of the correlation coefficient has been found to be 0.3400952 and the p-value of the test is 0.0156 < 0.05, which is significant at a 5% level of significance. So, we reject H0 at a 5% level of significance and conclude that there is a significant correlation between age and AZ distance. In graphical representation using a line graph with a trendline, a clear upward trend is evident in the length of the AZ distance with age (Figure 4).

## Discussion

Existing literature provides abundant data on the lower end of the esophagus. With an increased understanding of the pathological conditions affecting the lower end of the esophagus that are considered precancerous, this has become one of the most clinically significant areas in the body. Yet, the details and the precise anatomy of the region are not very clear. Several authors have studied the position of the Z line in relation to the GEJ (A) and the AZ distance corresponding to the CLE in normal subjects. In their study, Creamer et al. found the AZ distance to be in the range of 14-32 mm [17]. Bonibeck et al. found it to be in the range of 4-20 mm [16]. Liebermann-Meffert recorded a range of 9-21 mm [15], while Takubo et al. recorded a range of 0-10 mm [18]. In our present study, we found the AZ distance to be in the range of 0-20.2 mm.

Similarly, there was much variation in the mean of the AZ distance/length of CLE according to various studies. Studies based on autopsy found the length to be in a range from 10.4 mm [5] to 11 mm [6]. Simultaneously, radiology and endoscopic studies found this length to be 20 mm [7] and 15 mm [8], respectively. In our study, we found the mean AZ distance to be 12.4 mm with a standard deviation of ±5.3 mm, corroborating mostly with the previous cadaver-based studies.

The AZ distance corresponds to the length of the CLE and is crucial in defining BE. The length of CLE holds a significant correlation with intestinal metaplasia. Some authorities opine that even though long-segment BE is at a higher risk of developing carcinoma, a significant number of carcinomas are also seen to have developed in short-segment BE patients. They have attributed this to the imprecise measurement of BE in endoscopic procedures owing to the difficulty in identifying the exact point of transition from the esophagus to the stomach. At times this has also led to disagreement among endoscopists about the presence or absence of BE in the same patient [8]. An endoscopic study done on patients with a mean age of 48.15±10.90 years found that the patients with intestinal metaplasia had a CLE with a mean±SD length of 3.0±1.26 cm, while patients without metaplasia had a CLE length of 1.8±0.59 cm (p=0.001) [1]. In our study, the maximum length of CLE in normal subjects above 60 years was found to be 1.7± 1.99, which, therefore, falls well within the normal anatomical limit. Moreover, if we consider the length of the CLE in normal subjects, there was a mild increase in the measurements found during endoscopy when compared to cadaveric-based studies [5,6,8]. This discrepancy might represent a change in post-mortem anatomy or perhaps is due to the fact that the A, which is the true anatomical GEJ, is not possible to determine from the luminal side of the esophagus. Instead, the first appearance of gastric folds is usually taken as a landmark that may not coincide exactly with the A or the anatomical GEJ. Thus, defining the A, the true anatomical GEJ becomes difficult during routine endoscopic procedures. Additionally, even though this difference in the AZ distance/length of CLE found in our cadaveric study compared to previous endoscopic studies is minimal, it can be significant while defining the actual length of the CLE. This difference in length may be critical while diagnosing BE and perhaps leaves scope to recognize and refine the diagnostic accuracy in determining the critical length of actual CLE based on the true anatomical GEJ. Further, in our study, it was seen that there was a significant correlation between the AZ distance/anatomical CLE and the age of the individual. This can be of utmost importance while diagnosing BE in elderly patients.

Limitations of our study

A cadaveric study like ours might not be representing the anatomy of the gastroesophageal junction in live subjects. Correlating BE through AZ distance in cadavers lacks predictors like gastro-oesophageal reflux, which are usually considered in studies on live subjects. Correlating the study with the histology of the CLE can establish further evidence. With a bigger sample size, the study can have a better impact. We were not able to measure the entire length of the esophagus for its correlation with the AZ distance. This is one point where the study can be improved upon. 

## Conclusions

An accurate description and definition of the columnar-lined esophagus in the lower end is of paramount importance. The diagnosis of BE relies heavily on the proximal shift of the Z line from its normal anatomical position. The anatomical GEJ may not correspond to endoscopically measured GEJ. Therefore, the endoscopically measured CLE does not represent the true anatomical CLE or the AZ distance. Our present study suggests that the length of the CLE increases with age. This observation of ours creates a scope to revise/revisit the diagnostic criteria based on CLE length in accordance with the age of the individual.
